# Bibliographical Notices

**Published:** 1854-04

**Authors:** 


### EDITORIAL DEPARTMENT.

### BIBLIOGRAPHICAL NOTICES.

*Homœopathy, its Tenets and Tendencies, Theoretical, Theological and Therapeutical.* By James Y. Simpson, M. D., F. R S. E., Professor of Midwifery in the University of Edinburg, &c. &c. First American from the third Edinburg edition. Philadelphia. Lindsay & Blakiston, 1854.

There are certain falsehoods so glaring and so monstrous that it requires an enormous amount of self-control to treat them with any thing like gravity or sobriety. If homœopathy does not belong to this category, we must confess we do not know how to classify it.

When a man comes before the world with the grave announcement that a certain drug, in the proportion of about the quadrillionth or the decillionth of a grain, will produce paralysis, sleeplessness, humming in the ears, embarassed speech, shocks at the pit of the stomach, palpitation of the heart, and a great variety of similar severe symptoms, and claims this as the result of numerous and carefully repeated experiments, we naturally suppose that he is speaking of some terribly energetic poison, far exceeding in its destructive powers any deadly agent of which we have any knowledge. Aconitina, the effective principle of monkshood, produces its unpleasant effects in the dose of the seventy-fifth to the fiftieth of a grain, and that is the most energetic of the poisons yet introduced into the materia medica. But these are gigantic doses compared to the largest of this frightful agent. They bear the same proportion to it that twenty-five thousand millions of millions of tons do to them. What unheard of agent can this be? asks the alarmed reader, trembling lest an impalpable grain of it floating through the air might reach and annihilate him. What can he think then of the sanity of the discoverer, when told that the tremendous potency is no more than *common salt*, of which "a single tear drop contains millions of times more than would suffice for the treatment of all the individuals of the human family who have ever had an existence?" We frankly confess such assertions as these to be altogether too much for our gravity.

Dr. Simpson, however, has put a strong control upon his feelings, and has gone into the examination of this monster delusion with a gravity and industry beyond all praise. Dreading lest he might be accused of exaggeration and misrepresentation, he quotes the writings of renowned homœopathists, from Hahnemann down to the present day. Such monstrous and incredible statements, we confess, we were not prepared for, although perfectly aware of the general absurdity of homœopathic doctrines.

The doctor first considers the relations of homœopathists to physicians, and vindicates the course of the British universities and associations in refusing to recognize these pernicious quacks. He then goes into a consideration of infinitesimal doses, psorism, dynamization, olfaction, &c. The absurd experience argument is then taken up, its fallacy shown, and its applicability to all forms of quackery exposed. Last of all the doctrine of ***similia similibus*** is taken up.

The book is a little diffuse, but it rips up the follies of homœopathy, and discloses various rascally maneuvers on the part of its practitioners. Altogether it is the most complete ***exposé*** of this species of charlatanism we have yet read, and we commend it not only to the profession, but the public generally.

---

*Homœopathy fairly Represented; a Reply to Professor Simpson's "Homœopathy" misrepresented.* By William Henderson, M. D., Professor of General Pathology in the University of Edinburgh. First American, from the last Edinburgh edition. Philadelphia. Lindsay & Blakiston.

It was certainly a great misnomer in Professor Henderson to call this a ***reply*** to the book last noticed. He studiously avoids Professor Simpson's strong points and makes up for his deficiency in logic by copious personality. His style is theatrical, and manifestly aimed at the general public. There is a spite and venom about the book very appropriate in a man, who, feeling himself worsted in argument and unable to reply, vents his anger in impotent and unmeaning abuse.

He does not pretend to deny Doctor Simpson's statements, but writes a biography of Hahnemann with a running commentary on his doctrines and an occasional allusion to the doctor's book. He dwells largely on the numerical method of observation and the results obtained at Fleischmann's hospital at Vienna, but takes good care to avoid Dr. Simpson's arguments to show the fallacies of these deductions.

The numerical method, after all and at the best, can only afford approximative results. It is impossible to gain any satisfactory knowledge of pathology or therapeutics from it. Of what avail is it to us to know that in 10 out of 12 cases of a disease there were certain anatomical changes, unless we know the symptoms which accompanied them. So too in regard to therapeutics; it is idle to talk about averages of treatment. We must know the adaptation of means to ends, or we know nothing.

Take this very pneumonia about which Dr. Henderson makes such an ado. What avail the tables of Grisolle, or Lewis, or Dietl, or Fleischmann? What physician, who has ever stood at the bedside of a patient afflicted with pneumonia can be persuaded of the inutility of the lancet. You might as well try to persuade him that light was unnecessary to vision. The fault of all these tables is that they do not take into account the condition of the patient. One can very easily understand that the lancet may be inefficacious in fully formed pneumonia, but jugulate immediately the same disease while just setting in.

Dr. H. makes some allusion to animal chemistry. This is too absurd. Homœopathy stands about as much chance before physiological chemistry as the paper on which its doctrines are written does in the jet of an oxyhydrogen blowpipe.

*The Georgia Blister and Critic, for March,* 1854. Edited by H. A. Ramsay, M. D.

The journal with this ominous title hails from Atlanta, Georgia, and is, we are told, devoted to the development of southern medical literature and the exposition of the diseases and physical peculiarities of the negro race.

*The American Medical Monthly.* Published by G. P. Putnam, N. York.

We have received three numbers of this journal, which is a new claimant for the patronage of the medical profession. It is well conducted. Its original department is excellent and its selections judicious. We wish it success.

